# A Randomized Controlled Trial of Puncturing and Bloodletting at Twelve Hand Jing Points to Treat Acute Carbon Monoxide Poisoning as Adjunct to First Aid Treatment: A Study Protocol

**DOI:** 10.1155/2015/827305

**Published:** 2015-08-03

**Authors:** Ying Yue, Xingfang Pan, Sai Zhang, Jun Jin, Wei Wang, Dongqiang Wang, Dexin Han, Guirong Wang, Qunliang Hu, Jingqing Kang, Shasha Ding, Yi Yang, Huaien Bu, Yi Guo

**Affiliations:** ^1^Clinical Teaching and Training Department, Tianjin University of Traditional Chinese Medicine, Tianjin 300193, China; ^2^College of Acupuncture-Moxibustion and Tuina, Tianjin University of Traditional Chinese Medicine, Tianjin 300193, China; ^3^Neurosurgery and Neurology Hospital, Affiliated Hospital, Logistics University of the Chinese People's Armed Police Force, Tianjin 300162, China; ^4^International Education College, Tianjin University of Traditional Chinese Medicine, Tianjin 300193, China; ^5^Department of Integration Traditional and Western Medicine, Tianjin First Central Hospital, Tianjin 300192, China; ^6^Emergency Department, Wuqing Affiliated Hospital, Tianjin University of Traditional Chinese Medicine, Tianjin 301700, China; ^7^Emergency Department, Tianjin Binhai New Area Dagang Hospital, Tianjin 300270, China; ^8^Research Centre for Standardization of Acupuncture-Moxibustion, School of Language and Culture, Tianjin University of Traditional Chinese Medicine, Tianjin 300193, China; ^9^College of Traditional Chinese Medicine, Tianjin University of Traditional Chinese Medicine, Tianjin 300193, China

## Abstract

*Background*. Acute carbon monoxide poisoning (ACOP) is a significant cause of morbidity and mortality in many countries. Twelve Hand Jing Points (THJP) have been believed to be effective to treat all kinds of emergency calls in traditional Chinese medicine (TCM) for more than 3000 years. This randomized controlled trial (RCT) is designed to evaluate the effectiveness of THJP in curing acute carbon monoxide poisoning in first aid treatment. This paper reports the protocol of the trial. * Methods/Design. *This RCT is a multicenter, randomized, controlled study undergoing in China. The compliant patients are divided into the bloodletting group and standard of care group. With first aid treatments given to both of the groups, the bloodletting group is bleeding at THJP upon being hospitalized. Primary outcomes and secondary outcomes will be measured and compared between these two groups. Before treatment, immediately after treatment, and 30 minutes, 1 hour, and 4 hours after treatment, patients' basic vital signs and state of consciousness were observed. Before treatment and 1 and 4 hours after treatment, carboxyhemoglobin concentration in venous blood samples was detected.* Discussion. *The objective of this study is to provide convincing evidence to clarify the efficacy and safety of THJP for early treatment of acute carbon monoxide poisoning.

## 1. Background

Acute carbon monoxide poisoning (ACOP) is a significant cause of morbidity and mortality in many countries, the rate of the largest number of poisoning accidental poisoning of life and occupational poisoning [1, 2]. It may account for more than half of the world fatal poisoning [3].

ACOP is rather common in winter of China. CO diffuses readily within the alveoli and has far greater affinity than oxygen for essential biological compounds such as hemoglobin and cellular oxidative enzymes. Thus, CO poisoning causes cellular hypoxia. However, the mechanisms of its complications remain unclear [4–6].

Although hyperbaric oxygen therapy (HBO) produces a larger reduction in carboxyhemoglobin half-life, its benefit is that risk ratio is still debated [7]. From the current research on acute carbon monoxide poisoning, researches on first aid measures right after the onset are few, but they can effectively solve hypoxia and protect the brain tissue [5].

Twelve Hand Jing Points are of the Chinese traditional medical first aid measures. They have been believed to treat all kinds of emergency for more than 3000 years. Previous studies have shown that THJP may be effective in the first aid treatment of acute carbon monoxide poisoning but have not provided conclusive evidence of THJP bloodletting for acute carbon monoxide poisoning [8]. Previous animal studies indicated that compared with the control group, the indicator of carboxyhemoglobin concentration and awaking time both significantly decreased with the mice receiving the THJP treatment with acute carbon monoxide poisoning [9, 10]. For this study, the objective is to clarify the efficacy and safety of THJP in first aid treatment of acute carbon monoxide poisoning.

## 2. Methods and Design

### 2.1. Design

**Figure 1:**
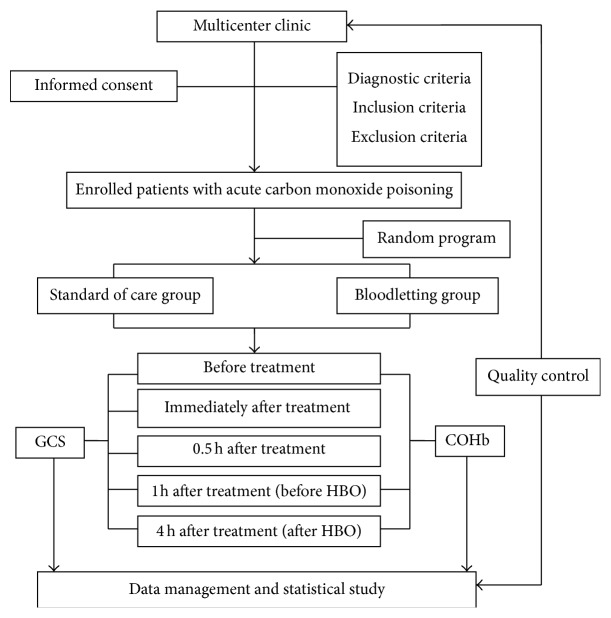
Trail flow chart.

This is a multicenter randomized controlled trial, enrolling both men and women with acute carbon monoxide poisoning in China. This study will be conducted at Neurosurgery and Neurology Hospital, Affiliated Hospital, Logistics University of the Chinese People's Armed Police Force; Wuqing Affiliated Hospital, Tianjin University of Traditional Chinese Medicine, and Tianjin Binhai New Area Dagang Hospital. The patient or the patient's family should write the informed consent for the study. The compliant patients according to visit order, by the method of central random [11], are divided into the bloodletting group and standard of care group. A designated researcher will provide patient groups, and acupuncturists do not know patient groups before treatment. The acupuncturists only know the group assignment prior to the treatment (see Figure 1).

This trial is financed by the National Natural Science Foundation of China (number 81303023). The research protocol has been approved by the Clinical Trial Ethics Committee of Affiliated Hospital of Logistics University of Chinese People's Armed Police Forces (trial registration number: ChiCTR-IOR-15005797).

### 2.2. Patients

#### 2.2.1. Study Population

Our study will include patients with acute carbon monoxide poisoning. To ensure the precision of results of this trial, patients who meet the following eligible criteria will be included in this study.

#### 2.2.2. Inclusion Criteria

1. Meeting the diagnostic criteria of GBZ23-2002 in acute carbon monoxide poisoning.
2. The course of disease is within 6 hours.
3. After the poisoning patients with disturbance of consciousness.

#### 2.2.3. Exclusion Criteria

1. Patients with delayed encephalopathy after acute carbon monoxide poisoning, cerebral hernia.
2. Liver and kidney function in previous severe abnormality.
3. Pregnant women, diabetes, and mental patients.
4. The blood coagulation dysfunction.
5. Doctors belief that there are some cases not suitable for inclusion.
6. Being not followed up.

### 2.3. Intervention

#### 2.3.1. Standard of Care Group

The standard of care group will receive general first aid treatment including hyperbaric oxygen therapy (HBO) about 45–60 minutes, citicoline 1 g intravenously once daily, and naloxone 0.4 mg intravenously every 12 hours.

#### 2.3.2. Bloodletting Group

The bloodletting group will receive general first aid treatment, the same as control group. Meanwhile, the bloodletting group will be treated with THJP on admission immediately before hyperbaric oxygen therapy. The order of puncture and bloodletting should be set. In this study, we decide to puncture and bloodlet the left hand first and then the right hand in the order of Shaoshang (LU11), Shangyang (LI1), Zhongchong (PC9), Guan Chong (TE1), Shaochong (HT9), and Shaoze (S11). And the amount of bloodletting at each point is one drop. At the same time, we choose a unified model needle. The location of acupoints is as follows [12].

*Shaoshang (LU11)*. It is on the thumb, radial to the distal phalanx, 0.1 F-cun proximal-lateral to the radial corner of the thumb nail, at the intersection of the vertical line of the radial border and the horizontal line of the base of the thumb nail. 

*Shangyang (LI1)*. It is on the index finger, radial to the distal phalanx, 0.1 F-cun proximal-lateral to the radial corner of the index fingernail, at the intersection of the vertical line of the radial border of the fingernail and the horizontal line of the base of the index fingernail. 

*Zhongchong (PC9)*. It is on the middle finger, at the centre of the tip of the middle finger, 0.1 F-cun proximal-lateral to the radial corner of the thumbnail, at the intersection of the vertical line of the radial side of the nail and the horizontal line of the base of the finger nail. 

*Guan Chong (TE1)*. It is on the ring finger, ulnar to the distal phalanx, 0.1 F-cun proximal to the ulnar corner of the fingernail, at the intersection of the vertical line of the finger side of the nail and the horizontal line of the base of the finger nail. 

*Shaochong (HT9)*. It is on the little finger, radial to the distal phalanx, 0.1 F-cun proximal-lateral to the radial corner of the little finger nail, at the intersection of the vertical line of the radial border of the nail and the horizontal line of the base of the little finger nail. 

*Shaoze (S11)*. It is on the little finger, ulnar to the distal phalanx, 0.1 F-cun proximal-medial to the ulnar corner of the little finger nail, at the intersection of the vertical line of ulnar border of the nail and the horizontal line of the base of the little finger nail.

### 2.4. Outcome Measures

In this study, there are two primary outcomes and four secondary outcomes. These are presented in Table 1. Any adverse event resulting from THJP or adverse drug reaction will be recorded. Safety evaluation for THJP will be based on events which include hematoma, syncope, local infection, and any feelings of discomfort. Any adverse event resulting from THJP or adverse drug reaction to THJP will be recorded.

#### 2.4.1. Primary Outcomes

Patients' states of consciousness (in Glasgow consciousness disorder table of GCS score) are observed at the following time points (before treatment, immediately after treatment, and 30 minutes, 1 hour, and 4 hours after treatment) in two groups. And patients' carboxyhemoglobin (COHb) concentration of venous blood samples is detected at the following time points (before treatment and 1 hour and 4 hours after treatment) in two groups.

#### 2.4.2. Secondary Outcomes

Patients' basic vital signs including temperature (*T*), pulse (*P*), respiration (*R*), and blood pressure (BP) are observed at the following time points (before treatment, immediately after treatment, and 30 minutes, 1 hour, and 4 hours after treatment) in two groups.

### 2.5. Sample Size and Statistical Analysis

Sample size will be based on the primary outcome. According to our pilot trial and review of the literature [8], the average defecation rate of patient with COHb was 22.40% with a standard deviation of 3.65% and the average defecation rate of patient with COHb was 17.78% with a standard deviation of 5.68%. To detect COHb% difference, we need a sample size of 30 in each group (*α* = 0.05, *β* = 0.10) [13]. To allow 5% for loss to follow-up, our proposed trial requires 62 patients (31 for each group).

Statistical analysis of all data will be done by Department of Public Health Statistics, which is blinded to this trial, using SPSS 19.0 software packages to analyze the data. All data are provided as mean ± standard deviation. The level of significance was set a *P* value less than 0.05. All of the analyses will be based on both an intention-to-treat analysis and a treated-per-protocol analysis. The intention-to-treat population was defined as the patients who are randomized and received at least one treatment session. The per-protocol population was defined as the patients who have no major protocol violations in the set. Both results of analysis will be compared to check whether the results are consistent.

## 3. Discussion

According to TCM theory, the body is regarded as a whole. It is composed of internal and external organs. These organs connect with each other by special ways called meridians [14]. Twelve Hand Jing Points are located at the end of fingers like the source of spring. Jing points are compared to the underground spring and the pulses here are so small.

*Well* or* Jing* is like underground water. It is described that the pulse is so small. The body of each of the twelve meridians has a well hole; they are also called* Jing Points*. Puncturing and bloodletting at Twelve Hand Jing Points are traditional Chinese medicine first aid measures used in various types of aid for more than 3000 thousand years in history. Some studies have indicated that acupuncture can play a therapeutic role in awaking consciousness disorders. From the clinical trials, we can see the potential for cerebral protection [8, 15–18]. However, this deduction is insufficient and may lead to bias because of the poor quality which is without a standardized acupuncture protocol. Therefore, we designed a multicenter randomized controlled protocol and expected to provide convincing evidence to clarify the efficacy of THJP for first aid treatment of acute carbon monoxide. Acupuncture is a nontoxic, economical intervention with minimal adverse effects [19], which has been shown to remain effective even for a few months after the therapy [20]. One of the limitations in this trial design is the nonblinding of the patients and acupuncturists. Due to the characteristics of THJP, it is impossible to blind the patients and acupuncturists. However, we develop a rigorous methodology in other aspects of the study.

## Figures and Tables

**Table 1:** Trial processes chart.

Times	Baseline		Treatment		
	−1	0	0.5 h	1 h	4 h
Patients					
Medical history	*√*				
Physical examination	*√*	*√*	*√*	*√*	*√*
Laboratory examination	*√*			*√*	*√*
Informed consent	*√*				
Randomization	*√*				
Intervention					
Standard of care group	General first aid treatment				
Bloodletting group	General first aid treatment + THJP				
*Outcome *					
primary outcomes					
GCS	*√*	*√*	*√*	*√*	*√*
COHb	*√*			*√*	*√*
Secondary outcomes					
T	*√*	*√*	*√*	*√*	*√*
P	*√*	*√*	*√*	*√*	*√*
R	*√*	*√*	*√*	*√*	*√*
BP	*√*	*√*	*√*	*√*	*√*
Trial evaluation					
Safety of hand pricking blood therapy	*√*	*√*	*√*	*√*	*√*
Adverse event	*√*	*√*	*√*	*√*	*√*
Reasons of drop-outs or withdrawals					*√*
Patient's compliance					*√*

## Trial Status

Twenty participants have been recruited.
